# Impact of Stomatal Density and Morphology on Water-Use Efficiency in a Changing World

**DOI:** 10.3389/fpls.2019.00225

**Published:** 2019-03-06

**Authors:** Lígia T. Bertolino, Robert S. Caine, Julie E. Gray

**Affiliations:** ^1^ Grantham Centre for Sustainable Futures, University of Sheffield, Sheffield, United Kingdom; ^2^ Department of Molecular Biology and Biotechnology, University of Sheffield, Sheffield, United Kingdom

**Keywords:** water-use efficiency, stomatal conductance, stomatal density and size, drought response, crops

## Abstract

Global warming and associated precipitation changes will negatively impact on many agricultural ecosystems. Major food production areas are expected to experience reduced water availability and increased frequency of drought over the coming decades. In affected areas, this is expected to reduce the production of important food crops including wheat, rice, and maize. The development of crop varieties able to sustain or improve yields with less water input is, therefore, a priority for crop research. Almost all water used for plant growth is lost to the atmosphere by transpiration through stomatal pores on the leaf epidermis. By altering stomatal pore apertures, plants are able to optimize their CO_2_ uptake for photosynthesis while minimizing water loss. Over longer periods, stomatal development may also be adjusted, with stomatal size and density being adapted to suit the prevailing conditions. Several approaches to improve drought tolerance and water-use efficiency through the modification of stomatal traits have been tested in the model plant *Arabidopsis thaliana*. However, there is surprisingly little known about the stomata of crop species. Here, we review the current understanding of how stomatal number and morphology are involved in regulating water-use efficiency. Moreover, we discuss the potential and limitations of manipulating stomatal development to increase drought tolerance and to reduce water loss in crops as the climate changes.

## Introduction

Changes in climate are already negatively affecting the yields of staple crops in agricultural areas around the world (Lobell et al., 2011; IPCC, 2014). As the globe continues to warm, changes in the hydrological cycles are, in general, increasing aridity and the incidence of droughts (Dai, 2013; Sherwood and Fu, 2014). Agriculture will need to adapt quickly to ensure that water is used more efficiently, while maintaining food security in a world where human population is rapidly growing. Sustainable and climate-smart management of water, land, and biodiversity will be important for achieving these objectives (discussed in Howden et al., 2007; Campbell et al., 2014; Lipper et al., 2014; Iglesias and Garrote, 2015). Moreover, the development of crop varieties that have improved water-use efficiency (WUE) under predicted future climates will also be critical (Flexas, 2016; Varshney et al., 2018). WUE can be estimated at different scales; at an agronomic level, it is described as the ratio of water used in crop production versus biomass or yield (Condon et al., 2004; Medrano et al., 2015). From a plant physiology point of view, and as it will be primarily addressed here, WUE is the amount of CO_2_ fixed in photosynthesis (*A*) relative to the amount of water vapor lost to the atmosphere (Willmer and Fricker, 1996; Condon et al., 2004; Bacon, 2004). As stomata play a fundamental role in regulating plant water use and carbon gain, they present a key target for improving WUE. Here, we review how changes in stomatal developmental traits can affect plant WUE and also drought tolerance.

Stomata are microscopic structures consisting of a pair of specialized guard cells that surround a central pore. They are found on aerial surfaces of most plants, providing access to mesophyll cells (Zeiger et al., 1987; Hetherington and Woodward, 2003). By actively adjusting guard cell turgor pressure, plants can alter stomatal pore aperture, thereby moderating gas exchange rates between the leaf interior and the atmosphere (Zeiger et al., 1987; Kollist et al., 2014). Increases in guard cell turgor pressure lead to a greater stomatal pore aperture, which enhances the rates of CO_2_ uptake for *A* and of water loss, *via* a process termed stomatal conductance (*g_s_*) (Condon et al., 2002; Hetherington and Woodward, 2003). On the other hand, reductions in guard cell turgor pressure lead to decreases in stomatal aperture and in *g_s_*. The signals governing the fluxes of CO_2_ and water to and from the plant mesophyll are highly coordinated, allowing plants to finely balance the need for carbon with the need to moderate water loss (Wong et al., 1979; Haworth et al., 2016; Sorrentino et al., 2016). This internal crosstalk is influenced by many environmental factors, including changes in temperature, light intensity, atmospheric CO_2_ concentration, air humidity, and soil moisture content (Farquhar and Sharkey, 1982; Schroeder et al., 2001; Mott, 2009; Assmann and Jegla, 2016; Chaves et al., 2016). For example, when water becomes limited, signals such as reduced hydraulic conductivity and increased abscisic acid (ABA) arise, causing guard cell turgor pressure decreases, which result in reduced stomatal aperture and *g_s_* (Schroeder et al., 2001; Mustilli, 2002; Tombesi et al., 2015; Bartlett et al., 2016; McAdam et al., 2016). These changes lead to an improved water conservation, but often at the expense of *A* (Flexas and Medrano, 2002). Conversely, when water is plentiful in the soil or air, guard cell turgor increases, leading to increases in stomatal pore aperture and in *g_s_*, with *A* also often increasing.

Over longer periods, external signals perceived by mature leaves can also lead to systemic responses that moderate stomatal development on the new leaf epidermis, resulting in changes in stomatal patterning (Casson and Gray, 2008; Casson et al., 2010; Pillitteri and Torii, 2012; Chater et al., 2014; Qi and Torii, 2018). Exposure of mature leaves to high CO_2_ or low light levels, for example, is known to cause reductions in stomatal density (*SD,* number of stomata per unit of area) and in stomatal index (*SI*, ratio of stomata to epidermal cells plus stomata, multiplied by 100) of new developing leaves (Lake et al., 2001; Miyazawa et al., 2006). Conversely, low CO_2_ and high light generally have the opposite effect. The impact of water availability on stomatal development is less understood, with mixed responses and differences among species being reported (Clifford et al., 1995; Xu and Zhou, 2008; Doheny-Adams et al., 2012; Sun et al., 2014; Zhao et al., 2015). In Arabidopsis, plants grown under water restriction do not show altered *SD*; however, reductions in stomatal size (*SS*, guard cell area, based on guard cell pair length and width) were observed (Doheny-Adams et al., 2012). These plastic modulations of number and size of stomata allow plants to adjust their stomatal pore area in response to the surrounding environment, ultimately affecting their maximum and minimum gas exchange.

**Figure 1:**
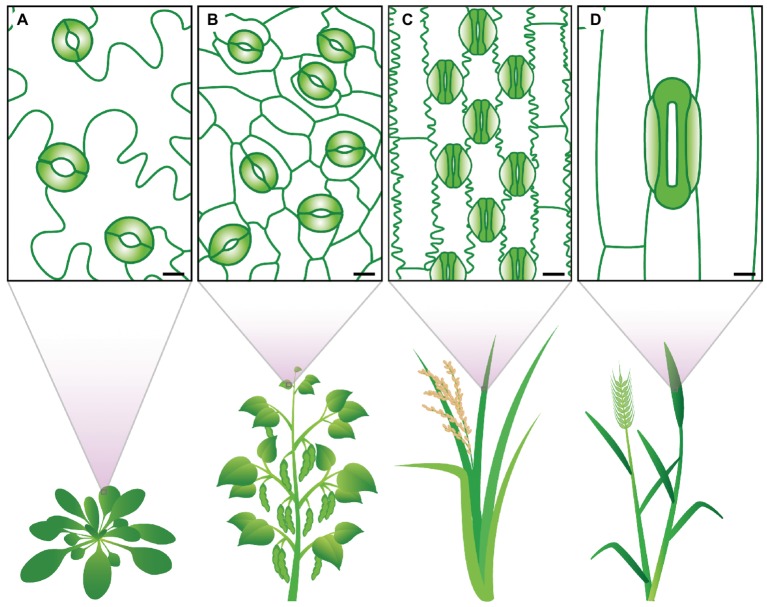
Stomatal traits vary between species. The eudicots **(A)** *Arabidopsis thaliana* and **(B)** *Phaseolus vulgaris* display kidney-shaped guard cells (colored in green). The grasses **(C)** *Oryza sativa* and **(D)** *Triticum aestivum* show dumbbell-shaped guard cells (solid green) and specialized subsidiary cells (light green gradient). Clear differences in stomatal size and stomatal density can be observed. Scale bars 10 μM.

Stomata exhibit a diverse range of shapes, sizes, and numbers across different plant species (Figure 1). There are profound differences in how the stomata of different groups develop and are patterned on the epidermis (Sack, 1994; Caine et al., 2016; Raissig et al., 2016; Rudall et al., 2017). Morphological differences include *SD*, *SS*, guard cell shape, and presence or absence of subsidiary cells. All of these parameters have the potential to influence stomatal movement and, consequently, plant *A*, *g*_*s*,_ and WUE. Over evolutionary time, various stomatal traits have altered, potentially aiding in adapting plant species to new environments (Taylor et al., 2012; Drake et al., 2013; Haworth et al., 2018). Eudicots, for example, typically have kidney-shaped stomata that are formed on the leaf epidermis without a pre-determined location (MacAlister et al., 2007; Pillitteri and Dong, 2013). While in monocots, stomata can either be kidney-shaped or, as with the grasses, be composed of dumbbell-shaped stomata with neighboring subsidiary cells, collectively termed a stomatal complex (Rudall et al., 2017). In grasses, stomatal development is constrained to the leaf base, with stomatal pores being formed in specified cell files adjacent to veins (Stebbins and Shah, 1960; Rudall et al., 2013; Hepworth et al., 2018).

Although stomatal behavior, patterning and morphology are important factors that contribute to WUE (Lawson and Blatt, 2014; Lawson and Vialet-Chabrand, 2019), relatively little is known about how targeted modifications of stomatal traits affect physiological responses in crop plants, especially in field experiments. Efforts to improve WUE have often led to decreases in yield (Flexas, 2016). By attempting to alter stomatal features to improve water conservation, reductions in *g_s_* may arise, potentially leading to detrimental effects on *A*, evaporative cooling, and plant fertility. However, recent findings suggest that under at least some greenhouse and controlled environment growth conditions, changing stomatal traits may improve WUE without such undesirable yield penalties (Yu et al., 2013; Hughes et al., 2017; Caine et al., 2019). While these studies are encouraging, they offer only a snapshot of how plants with modifications in stomatal features might perform. Here, we discuss our current understanding of how alterations in *SS*, *SD,* and stomatal morphology contribute to altered WUE and drought tolerance with particular emphasis on the latest advances in crop species.

## Variation in *SS* and *SD* Influences Gaseous Exchange and WUE

Dynamic adjustments to the opening degree of stomatal pores are responsible for regulating *g_s_* in the short term, allowing plants to quickly reduce water loss according to external cues (Farquhar and Sharkey, 1982). Over a longer term, anatomical adjustments, such as changes to *SS* and *SD*, can modify the range of *g_s_* by altering the maximum stomatal conductance (g*_smax_*) (Franks et al., 2009; Dow et al., 2014a). *G_smax_* refers to the maximal potential gas exchange in a state where all stomata are fully open. It is a theoretical estimate that is calculated using empirical stomatal anatomical measurements, including *SD*, stomatal pore depth (estimated as guard cell width), and maximum stomatal pore area (calculated based on pore length) (Franks and Beerling, 2009; Dow and Bergmann, 2014; Sack and Buckley, 2016). Despite operating *g_s_* normally being significantly lower than its maximum capacity (Fanourakis et al., 2015; McElwain et al., 2016), measured *g_s_* positively correlates with calculated *g_smax_* (Franks et al., 2009). Furthermore, it is suggested that adaptations in *g_smax_* allow plants to adjust their operating gas exchange rates while maintaining guard cells turgor pressure in an optimum state. This is believed to provide better stomatal sensitivity and rapid adjustment of aperture response (Franks et al., 2012; Dow and Bergmann, 2014). Therefore, although *SS* and *SD* are not the only variables determining leaf gas exchange, changes to these stomatal traits do permit plants to adjust both *A* and water use (Franks et al., 2009; Franks and Beerling, 2009; de Boer et al., 2011; Dow et al., 2014a).

Variation in size and density of stomata may arise due to genetic factors and/or growth under different environmental conditions. A negative correlation has frequently been suggested between these two stomatal traits. This inverse relationship has been observed in plastic developmental responses to changes in environment and also during long-term evolutionary adaptation (Dilcher et al., 2000; Ohsumi et al., 2007; Franks et al., 2009, 2012; Franks and Beerling, 2009; Doheny-Adams et al., 2012; Taylor et al., 2012; Sun et al., 2014; Fanourakis et al., 2015; de Boer et al., 2016; Dittberner et al., 2018). Analysis of herbarium and fossilized plant remains suggest that *SS* and *SD* have changed in response to atmospheric CO_2_ concentration over evolutionary time, probably to enable adjustments to *g_smax_* and CO_2_ diffusion into the leaf (Woodward, 1987; Dilcher et al., 2000; Franks and Beerling, 2009). In samples from periods when CO_2_ concentrations were low, a reduction in *SS* and an increase in *SD* have been observed. On the other hand, when atmospheric CO_2_ levels have been high, *SS* has increased and *SD* decreased. Such adaptive responses to CO_2_ are also found in many extant lineages; however, this is not always the case in all species surveyed (Casson and Gray, 2008; Haworth et al., 2013; Field et al., 2015).

Although various combinations of *SS* and *SD* can result in similar alteration to *g_smax_*, there are limitations as to how much of the epidermis can be patterned by stomata. First, other functionally important leaf structures such as veins and trichomes are also absolutely required. Second, stomata need to be spaced by at least one epidermal cell to function efficiently (Franks and Farquhar, 2007; Dow et al., 2014b). Therefore, changes to *SS* and *SD* are limited to a finite portion of the epidermis (Franks et al., 2009; Franks and Beerling, 2009; de Boer et al., 2016). In general, plants optimize *g_smax_* through investing in increases in *SD* coupled with reductions in *SS* (Franks and Beerling, 2009; de Boer et al., 2012). According to Franks and Beerling (2009) and Franks and Farquhar (2007), changes toward increased *SD* in combination with reduced *SS* could maintain or improve total pore area (due to increased *SD*) but can also provide a shorter diffusion path (due to the smaller pore depth), potentially resulting in improved gas exchange.

While small *SS* coupled with high *SD* often leads to a higher *g_smax_*, it is also possible for *g_smax_* to be reduced by a smaller *SS* alone. Decreases in *g_smax_* due to a smaller *SS* have been associated with higher water conservation, as reported for plants exposed to drought (Doheny-Adams et al., 2012) and ABA treatment (Franks and Farquhar, 2001). Smaller stomata are also associated with improved WUE in *Arabidopsis thaliana* (Dittberner et al., 2018); and rice varieties with smaller *SS* have the ability to strongly decrease *g_s_* under drought (Ouyang et al., 2017). Growth under low soil moisture conditions has been shown to cause a decrease in *SS* in several species (Xu and Zhou, 2008; Doheny-Adams et al., 2012; Sun et al., 2014; Zhao et al., 2015), but the effect on *SD* is less consistent (Clifford et al., 1995; Xu and Zhou, 2008; Doheny-Adams et al., 2012; Sun et al., 2014; Zhao et al., 2015). Stomatal size and density responses to vapor pressure deficit (VPD) are also variable. Under high VPD conditions, the woody angiosperm, *Toona ciliata*, displays smaller *SS* and higher *SD* (Carins Murphy et al., 2014), while tomato and sweet pepper show decreases in both stomatal traits (Bakker, 1991), and in poplar, changes in *SD* are dependent on CO_2_ concentration. (Miyazawa et al., 2006). Thus, changes in *SS* and *SD* in response to soil moisture or VPD appear to be specific to species and environmental variables (Bakker, 1991; Miyazawa et al., 2006; Xu and Zhou, 2008; Sun et al., 2014). Nutrient availability can also affect plant development. However, as described for soil moisture and VPD responses, adjustments in stomatal development in response to nutrient availability appear to be variable with no consistent response emerging (Gao et al., 2006; Sekiya and Yano, 2008; Yan et al., 2012; Sun et al., 2014; Hepworth et al., 2016).

## Are Small Stomata Faster?

Small stomatal size can provide a reduction in total leaf pore area and might also facilitate faster aperture response (Franks and Beerling, 2009; Drake et al., 2013; Lawson and Blatt, 2014). The higher cell surface area to volume ratio of smaller cells is believed to permit faster ion fluxes, leading to faster guard cell turgor changes and a more rapid *g_s_* response (Lawson and Vialet-Chabrand, 2019). This faster stomatal behavior in plants with smaller *SS* has been observed in response to changes in light intensity across species of *Banksia*, rainforest trees, and in cereal species with dumbbell-shaped guard cells (Drake et al., 2013; McAusland et al., 2016; Kardiman and Raebild, 2017). However, although rapid stomatal movements might help to maximize WUE under fluctuating light environments, this is unlikely to have much impact on water loss over long periods of water stress under field conditions.

*SS* is clearly not the only anatomical trait influencing stomatal behavior. The shape of guard cells and the presence of subsidiary cells are also suggested to impact on stomatal responses (as discussed in “Stomatal morphology and improved WUE” below). In addition, the distribution of stomata between leaf abaxial and adaxial surfaces may also affect plant responses to environmental stresses. For example, stomata on the abaxial surfaces of wheat leaves show a stronger decrease in *g_s_* than adaxial stomata when they are exposed to water stress (Lu, 1989), and abaxial and adaxial stomata of cotton show differing responses to light quality (Lu et al., 1993). Moreover, as shown by Elliott-Kingston et al. (2016), Haworth et al. (2018), and McAusland et al. (2016), simple differences in *SS* do not always correlate with stomatal speed, especially when comparing distant taxa. Comparisons of cultivars or mutants of the same species with altered *SS*, but similar *SD*, would improve our understanding of the effect of guard cell size on speed of stomatal movement and explore if there is potential for *SS* as trait for improving WUE. Although a correlation between *SS* and genome size has been documented (Beaulieu et al., 2008; Jordan et al., 2015; Monda et al., 2016), the genetic and molecular mechanisms regulating *SS* remain unstudied, which currently limits our understanding of this trait.

## Targeted Changes in *SD* Leading to Alterations in WUE

Reductions in *SD* also have the potential to constrain *g_s_* and transpiration (*E*), representing a shift towards a more conservative use of water. If not limiting *A* or evaporative cooling, this reduction in water loss should represent an advantage under low water availability scenarios. In comparison to *SS*, significant advances have been made in understanding the molecular signals regulating stomatal density and patterning, which allow the study of the physiological effects of altering *SD*. Stomatal development in *Arabidopsis thaliana* is controlled by a complex genetic network, of which the basic helix-loop-helix (bHLH) transcription factors SPCH, MUTE, and FAMA together with either ICE1/SCREAM (SCRM) or SCRM2 control the sequential cell fate transitions. Additionally, an intercellular signaling pathway that includes peptide ligands, leucine-rich repeat receptor kinases (LRR-RKs) and a MAPK cascade, regulates the activity of the bHLHs (reviewed in Bergmann and Sack, 2007; Vatén and Bergmann, 2012; Zoulias et al., 2018). This signaling network includes the secretory peptides EPIDERMAL PATTERNING FACTOR1 (EPF1), EPF2, and EPF-like 9. EPF1 and EPF2 both negatively regulate stomatal density, with EPF1 also preventing stomatal clustering (Hara et al., 2007; Hunt and Gray, 2009), while EPFL9 functions antagonistically to promote stomatal development (Hunt et al., 2010; Sugano et al., 2010; Lee et al., 2015).

In Arabidopsis, the overexpression of *AtEPF2* results in plants with particularly low *SD*. Despite a coupled increase in *SS*, these plants have significantly lower *g_s_* and *g_smax_*, with minor reductions in carbon assimilation, leading to an increase in intrinsic WUE (iWUE, estimated by *A*/g_s_ to water vapor) (Doheny-Adams et al., 2012; Franks et al., 2015). The large reduction in *SD* resulted in plants with improved tolerance to drought, without detrimental effects to uptake of nitrogen or phosphate (Hepworth et al., 2015, 2016). Improved plant drought responses were also achieved by Wang et al. (2016) in poplar plants overexpressing *PdEPF1*. Transgenic poplar lines showed a 28% reduction in *SD*, which led to a 30% decrease in *E*, despite an increase in *SS*. WUE and drought tolerance were improved in poplar plants with lower *SD*, which also showed relatively lower decreases in levels of *A* and biomass under water restricted conditions (Wang et al., 2016). The manipulation of another regulator of stomatal development, the subtilisin-like protease STOMATAL DENSITY AND DISTRIBUTION1 (SDD1), also leads to a significant alteration to *SD* in Arabidopsis, with overexpression of this gene reducing *SD* by 40% (Von Groll et al., 2002). This reduction was translated into a lower *g_s_* under high light intensities; however, *A* was compromised under some light conditions (Büssis et al., 2006). A more encouraging result was achieved by Yoo et al. (2010) by manipulating a transcriptional repressor of SDD1, GT-2 LIKE 1 (GTL1). GTL1 loss-of-function Arabidopsis mutants had higher *SDD1* expression resulting in lower *SD* and *g_s_*, without detrimental effects to the photosynthetic rates over a range of light levels. The lower water loss observed in the *gtl1* mutants significantly improved WUE, when water loss versus shoot dry weight was assessed. Taken together, these data indicate that it is possible to improve WUE by altering *g_smax_* and *g_s_* using genetic engineering tools. It is not fully understood, however, how severe reductions in *g_smax_* may limit short-term stomatal responses or whether adjustments to stomatal development in response to changes in environmental conditions would be affected in these genetically modified plants.

Although stomatal development in grasses differs from that of eudicots in various aspects, recent findings demonstrate that several components of the stomatal signaling pathway, including bHLH transcription factors (Liu et al., 2009; Raissig et al., 2016, 2017) and peptide signals controlling stomatal density, mediate similar events (Hughes et al., 2017; Yin et al., 2017; Caine et al., 2019). This has allowed researchers to begin to test the implications of targeted manipulations in stomatal density in grasses, a family of plants that comprises many important food crops. Research on barley and rice (further discussed below) shows that the overexpression of *EPF1* can result in improved WUE without yield penalty, despite in some cases small reductions in photosynthetic rate under well-watered conditions (Hughes et al., 2017; Caine et al., 2019). Interestingly, in both crops, an increase in guard cell size was not observed in plants with reduced *SD*, contrasting with that described for poplar and Arabidopsis above. These observations suggest that the response of *SS* to altered *SD* may be differentially regulated between monocots and eudicots.

## Stomatal Morphology and Improved WUE

The shape of guard cells and the presence or absence of subsidiary cells have implications for the mechanics and responsiveness of stomatal movement (Franks and Farquhar, 2007). Diversity in stomatal morphology is commonly observed across species and can be linked to adaptability to certain environments (Chen et al., 2017; Müller et al., 2017). In the grass family, for example, stomatal morphology has often been hypothesized to have contributed to successful diversification, particularly in habitats with fluctuating water availability (Hetherington and Woodward, 2003; Cai et al., 2017; Chen et al., 2017). In contrast to the two kidney-shaped guard cells observed in many species, grass species develop stomatal complexes formed by a pair of dumbbell-shaped guard cells, which are flanked by two paracytic subsidiary cells (Stebbins and Shah, 1960; Sack, 1994; Rudall et al., 2017; Hepworth et al., 2018; McKown and Bergmann, 2018). Several studies comparing stomatal opening and closing responses, between grasses and species with kidney-shaped stomata, suggest that grasses exhibit faster and more efficient stomatal regulation (Grantz and Zeiger, 1986; Vico et al., 2011; Merilo et al., 2014; McAusland et al., 2016; Haworth et al., 2018). The linear dumbbell-shaped guard cells require only small changes in volume to bring about stomatal opening and, consequently, to achieve a higher diffusible pore area (Hetherington and Woodward, 2003). The large and rapid responses of grass stomata are also related to the physical interaction between dumbbell-shaped guard cells and flanking subsidiary cells. Subsidiary cells are not only able to limit but also to accommodate guard cell movement, providing a mechanical advantage (Franks and Farquhar, 2007). They function by promptly supplying ions to guard cells, facilitating a reciprocal change in turgor pressure (Raschke and Fellows, 1971; Franks and Farquhar, 2007; Schäfer et al., 2018). This efficient osmotic flux aids rapid stomatal movement and therefore is believed to confer adaptive advantages to grasses.

Slow stomatal responses are proposed to lead to less efficient uptake of CO_2_ during stomatal opening and unnecessary water loss during stomatal closure (McAusland et al., 2016; Lawson and Vialet-Chabrand, 2019). Under particular environmental conditions (e.g., fluctuations in irradiance), plants with stomata which are highly responsive might achieve higher WUE. Recently, the absence of subsidiary cells was investigated in *Brachypodium distachyon* plants with a mutation in *BdMUTE*, an ortholog of an Arabidopsis bHLH gene. Mutant plants lacking subsidiary cells failed to open guard cells as widely as control plants and also showed slower stomatal responses to changes in light intensity, further suggesting that subsidiary cells are integral for efficient stomatal functioning in grasses (Raissig et al., 2017). The receptor-like proteins PANGLOSS (PAN) 1 and PAN2 are also integral for the formation of subsidiary cells, primarily by enabling subsidiary mother cells to polarize in the correct orientation to guard mother cells during stomatal development (Cartwright et al., 2009; Zhang et al., 2012; Sutimantanapi et al., 2014). Defective *pan1* and *pan2* mutants and *pan1/pan2* double mutants have misshapen subsidiary cells, which could impact on stomatal responsiveness; however, it is not known whether gas exchange is affected in these mutant plants. Despite the relatively recent discoveries of MUTE and PAN proteins in grasses, there are still many unanswered questions in relation to subsidiary and guard cell interactions, especially in non-grass species, which show a diversity of stomatal complex morphologies, with different numbers and positions of subsidiary cells (Rudall et al., 2017). Further study of how the diversity of stomatal complex morphologies affects plant physiology could improve our understanding of how these features might contribute to improved WUE.

## Genetic Manipulation of Stomatal Development in Crops, the Implications for WUE, and Drought Responses

An increasing number of genetic resources are enabling researchers to test whether targeted alterations in stomatal development can improve WUE and drought tolerance in crop species (Winter et al., 2007; Goodstein et al., 2012; Yin et al., 2017). Although results are yet to be demonstrated in the field, in overexpressing orthologs of Arabidopsis *SDD1* in maize and tomato, respectively, Liu et al. (2015) and Morales-Navarro et al. (2018) have been able to reduce leaf *SD*, leading to reduced water consumption and improved drought tolerance in both crops, as well as improved WUE in maize. Similar results were achieved in barley by overexpressing *HvEPF1* (Hughes et al., 2017). With approximately 50% reduction in *SD* and shorter guard cells, under drought conditions, transgenic barley lines were able to retain higher levels of soil water content. These plants were able to avoid water stress for longer periods, showing drops in photosystem II activity 4–5 days later than the control plants. Carbon isotope analysis suggested that plants with reduced *SD* had improved WUE under the water stress treatment, and despite small reductions in *A*, no detrimental effects on plant growth or yield were observed (Hughes et al., 2017). Similarly, overexpression of rice *OsEPF1* resulted in rice plants with improved WUE (Caine et al., 2019). Two genetically modified lines, one with moderate (~58%) and the other with severe (~88%) reductions in *SD*, had improved water conservation during the vegetative stage, using 42% and 38% less water, respectively, than the control.

An opposite effect on stomatal development was created by overexpressing the maize gene *SHORTROOT 1* (*ZmSHR1*) in rice, leading to higher *SD* and in some cases reduced *SS* (Schuler et al., 2018). Despite the changes in stomatal properties, neither *A* nor *g_s_* was significantly different from controls suggesting that increased *SD* neither positively or negatively impacted on gaseous exchange. In this particular study, WUE was not reported, but based on *A* and *g_s_* values, alterations seem unlikely. Given the predicted temperature increases for the coming century, however, crop plants with more stomata and potentially increased gas exchange capacity may be important in mitigating the effects of heat stress through increased transpiration-mediated cooling.

While most of the crop studies discussed above have characterized drought and photosynthetic performance, to better understand how crops with altered *SD*, *SS*, or function might perform under future climate scenarios, it is important to consider the combinatory effects of multiple abiotic factors. Of particular importance are the predicted reductions in water availability, increasing atmospheric CO_2_, concentration, and increasing temperature. While reduced water availability and elevated CO_2_ often result in stomatal closure leading to reduced *g_s_*, increased temperature might have the opposite effect, forcing stomata to open to mitigate the effects of overheating (Zhou et al., 2007; Chaves et al., 2016; Caine et al., 2019). This essentially means that in future climates, if plants are going to conserve water, they may be less able to prevent overheating, possibly leading to photoinhibition, leaf damage, and reduction in yields.

**Figure 2:**
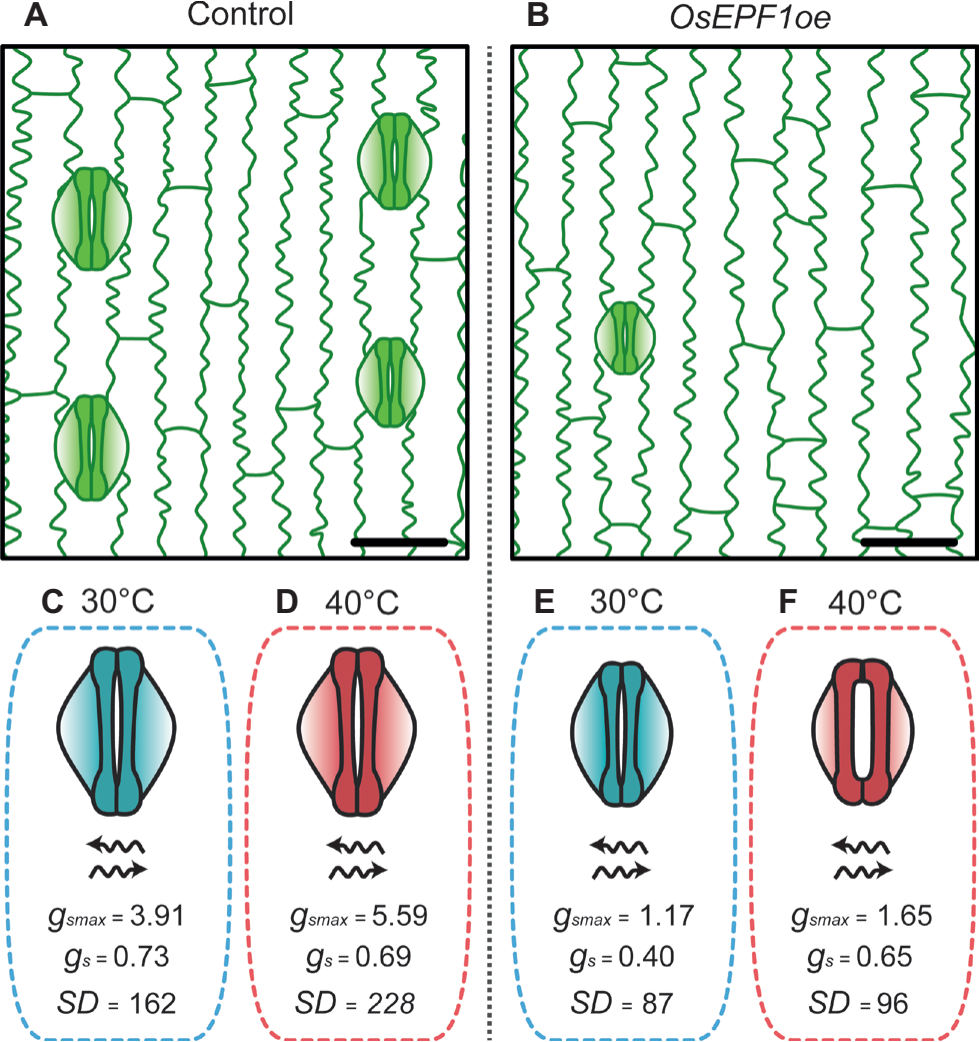
*OsEPF1oe* rice plants with reduced stomatal density and size are able to maintain high rates of gas exchange under heat stress conditions by opening their stomatal pores (adapted from Caine et al., 2019). Epidermis of **(A)** non-transgenic control and **(B)** *OsEPF1oe* plants grown at 30°C, bars = 25 μM. Stomata of control plants grown at **(C)** 30°C and **(D)** 40°C. Control plants show increases in stomatal density and in maximum leaf stomatal conductance average values under high temperature conditions. *OsEPF1oe* plants grown at **(E)** 30°C and **(F)** 40°C. Transgenic line shows an increase in stomatal aperture at 40°C, reaching similar *g_s_* levels as control plants, despite lower maximum stomatal conductance. Units: *g_smax_* and *g_s_* = mol m^−2^ s^−1^, *SD* = mm^−2^.

This trade-off between WUE and evaporative cooling was recently investigated in the *OsEPF1* overexpressing (*OsEPF1oe*) rice. Under well-watered conditions at high temperature (40**°**C), plants with substantially reduced *SD* (and *SS*) exhibited increased *g_s_*, reaching similar rates as control plants. The increase in gas exchange rates were seemingly achieved through regulation of stomatal apertures (Figure 2), with the trade-off being a loss of superior WUE relative to control plants (Caine et al., 2019). However, at 40**°**C, *OsEPF1oe* plants with severe *SD* reductions showed enhanced survival rates under drought stress, perhaps because of their improved soil water conservation under these conditions. Although these responses suggest that having low *SD* with reduced *SS* could be beneficial at very high temperature, at present the operational dynamics of stomata when temperature exceeds 40**°**C are not well understood. Modeling of *g_smax_* suggested that despite the much reduced *SD*, *OsEPF1oe* plants still use only up to 40% of their theoretical maximum gas exchange capacity at 40**°**C, but the actual level of *g_s_* (and *A* and WUE) at more extreme temperatures remains untested (Caine et al., 2019). These results raise a number of questions regarding the physiological behavior of these reduced *SD* plants. Firstly, will plants with fewer, smaller stomata be capable of continuing to increase *g_s_* to maintain water flow and *A* at extreme temperatures, and will this be at the expense of WUE? If so, will plants with the lowest *SD* be less water-use efficient than plants with higher *SD* at very high temperatures in order to maintain cooling? The answers to such questions are critical to understand if targeted *SD* reductions are to be an effective tool to improve rice production in areas where drought and high temperatures are predicted to become more prevalent.

## Other Potential Stomatal-Related Targets to Improve WUE

The central position of stomata in the gas exchange process makes them an obvious target for improving WUE; nonetheless, the manipulation of other processes with potential for improving plant carbon and water relations has also been investigated (Lefebvre et al., 2005; Taylor et al., 2011; Ort et al., 2015; Kromdijk et al., 2016; Głowacka et al., 2018). Alterations in mesophyll conductance (*g_m_*), for example, can have a great impact in *A*, and its coregulation with *g_s_* is essential for plant WUE. Indeed, it has been suggested that increases in *g_m_* coupled with decreases in *g_s_* could improve WUE, without the potential detrimental impacts in *A* and yield (Flexas et al., 2013).

Moreover, stomata are not the only structures on the epidermis to prevent water loss – trichomes, the cuticle, and cuticular waxes are also important (Guo et al., 2016; Ichie et al., 2016; Bi et al., 2017; Zeisler-Diehl et al., 2018). While research into crop plant stomata is of long-standing (Teare et al., 1971; Gay and Hurd, 1975; Liao et al., 2005; Ohsumi et al., 2007), new approaches are looking at drought-tolerant relatives of crops or desert-growing species for novel ways to increase WUE. For example, by crossing the wild drought tolerant tomato relative, *Solanum pennellii* (which has abundant trichomes), with the cultivated species *Solanum lycopersicum*, Galdon-Armero et al. (2018) showed that the coordinated development of trichomes and stomata may be a key tool for enhancing WUE in crops. It was found that the plants with the best WUE were those with the highest ratio of trichomes to stomata. One possible explanation for this is that plants with fewer stomata and abundant trichomes have a more significant boundary layer, thus creating a greater resistance to diffusion of water from the leaf (Galdon-Armero et al., 2018). Another example of increased resistance to diffusion of water due to adaptations to the epidermis has also recently been reported in the desert crop, date palm (*Phoenix dactylifera*) (Müller et al., 2017). In this study, wax chimneys were detected on the cuticle that encircled stomata, which like trichomes, prevented excessive water loss, thereby potentially improving WUE. Further investigations exploring how stomata and other epidermal structures jointly contribute to regulate WUE may be a critical piece in the jigsaw of preserving water and negating drought. Indeed, the recent discovery of the *Fused Outer Cuticular Ledge1* stomata gene in Arabidopsis may help facilitate such studies (Hunt et al., 2017).

## Conclusion

The knowledge relating to the genetics underpinning stomatal development and physiology in both Arabidopsis and crop species has advanced substantially, with noticeable advancements made in improving WUE. However, there are still many questions to answer, of particular importance is how *SS* is regulated at the genetic level and why do *SS-SD* responses vary so much between species. In addition to this, understanding how stomatal complex architecture is modified and how ion fluxes are directed between guard and subsidiary cells at the genetic level is the key area where further advances in knowledge are required. In crops, recent studies are showing that engineering plants to reduce stomatal number may be an effective tool to improve plant WUE and drought tolerance without yield reductions. Of course, as modified plants have typically been evaluated in laboratory conditions, it is still necessary to answer how such plants might perform in the real world. In a field context, many other environmental variables and stressors will impact on performance. Additional studies are necessary to understand how plants with altered stomatal development will respond to multiple stresses in different developmental phases. Moreover, combining changes in stomatal traits with other alterations associated with improved water relations, such as modifications to the leaf epidermis, photosynthesis, *g_m_*, and root growth, among others, could further benefit plant WUE and drought tolerance under future predicted climate scenarios.

## Author Contributions

LB and RC wrote the paper. LB designed the figures. JG provided advice and comments. All authors read, commented on, and approved this version of the manuscript.

### Conflict of Interest Statement

The authors declare that the research was conducted in the absence of any commercial or financial relationships that could be construed as a potential conflict of interest.
